# Association between metabolic syndrome severity score and coronary artery disease complexity assessed by the SYNTAX score in patients with chronic coronary syndrome

**DOI:** 10.3389/fcvm.2026.1907964

**Published:** 2026-10-28

**Authors:** Mehmet Vatansever, Ahmet Özderya, Fatma Yılmaz Tulumcu, Yılmaz Sezgin, Serap Özer Yaman

**Affiliations:** 1 Department of Family Medicine, Trabzon University, Trabzon, Türkiye; 2 Department of Cardiology, Trabzon University, Trabzon, Türkiye; 3 Department of Biochemistry, Trabzon University, Trabzon, Türkiye

**Keywords:** cardiovascular risk, chronic coronary syndrome, coronary angiography, coronary artery disease, metabolic syndrome severity score, syntax score

## Abstract

**Background:**

Metabolic syndrome represents a cluster of interrelated cardiometabolic abnormalities associated with coronary artery disease. However, conventional definitions evaluate metabolic syndrome categorically and may not adequately reflect its cumulative severity. The primary objective of this study was to assess whether the metabolic syndrome severity Z-score (MetS-Sz) was independently associated with coronary artery disease complexity, as quantified by the SYNTAX score, in patients with chronic coronary syndrome.

**Methods:**

This single-center retrospective cross-sectional study included 156 patients with chronic coronary syndrome who underwent coronary angiography. MetS-Sz was calculated using age-, sex-, race-, and body mass index-based metabolic variables, whereas coronary lesion complexity was assessed using the SYNTAX score. Participants were categorized into lower (MetS-Sz < 0) and higher (MetS-Sz ≥ 0) MetS-Sz groups, corresponding to values below and at or above the reference population mean. Clinical, anthropometric, and biochemical characteristics were compared between groups. Correlation analysis and multiple linear regression were performed to evaluate the association between MetS-Sz and the SYNTAX score.

**Results:**

Of the 156 participants, 123 (78.8%) were men and 33 (21.2%) were women. Patients in the higher MetS-Sz group had significantly higher body mass index, triglyceride, total cholesterol, low-density lipoprotein cholesterol, glucose, diastolic blood pressure, and SYNTAX scores and lower high-density lipoprotein cholesterol levels than those in the lower MetS-Sz group (all *p* < 0.05). MetS-Sz was positively correlated with the SYNTAX score (*r_s_* = 0.675, *p* < 0.001). The multiple linear regression model was statistically significant (*F* = 22.84, *p* < 0.001) and explained 51.9% of the variance in the SYNTAX score (R^2^ = 0.519). MetS-Sz, low-density lipoprotein cholesterol, body mass index, triglyceride level, and smoking status were independently associated with the SYNTAX score, whereas diabetes and diastolic blood pressure were not significantly associated with it.

**Conclusions:**

Higher MetS-Sz was independently associated with greater coronary artery disease complexity in patients with chronic coronary syndrome. These findings suggest that assessment of cumulative metabolic burden using MetS-Sz may complement conventional cardiovascular risk evaluation. Prospective, multicenter studies are required to confirm its potential role in coronary risk stratification.

## 1 Introduction

Metabolic syndrome (MetS) is a cluster of interrelated cardiometabolic abnormalities, including central obesity, elevated blood pressure, hyperglycemia, hypertriglyceridemia, and reduced high-density lipoprotein cholesterol levels. These abnormalities frequently coexist and substantially increase the risk of type 2 diabetes mellitus and atherosclerotic cardiovascular disease (1–4). Although conventional MetS definitions are widely used in clinical practice, they classify individuals categorically according to the presence or absence of a predefined number of components. This approach may not fully capture the cumulative severity of metabolic dysfunction or the continuous nature of cardiometabolic risk (4–6).

The metabolic syndrome severity Z-score (MetS-Sz) was developed to provide a continuous assessment of metabolic syndrome severity by integrating multiple metabolic components while accounting for demographic characteristics such as age, sex, and race or ethnicity (7). Previous studies have demonstrated that higher MetS-Sz values are associated with an increased risk of incident diabetes, coronary heart disease, cardiovascular events, and mortality (8–12). Compared with the conventional binary definition of MetS, MetS-Sz provides a continuous measure of metabolic syndrome severity that allows examination of its association with coronary anatomical complexity. Metabolic disturbances contribute to the initiation and progression of coronary atherosclerosis through several interconnected mechanisms, including insulin resistance, endothelial dysfunction, chronic low-grade inflammation, oxidative stress, dyslipidemia, and hypertension (2, 13–15). However, the association between cumulative metabolic syndrome severity and the anatomical complexity of coronary artery disease remains insufficiently understood. Evaluation of this relationship may be clinically relevant because anatomical lesion complexity influences both prognosis and revascularization strategy in patients with coronary artery disease.

The SYNTAX score is an angiographic scoring system developed to quantify the extent and complexity of coronary artery disease. It incorporates the number, location, and morphological characteristics of coronary lesions and is widely used to support clinical decision-making regarding percutaneous coronary intervention and coronary artery bypass grafting (16–18). Higher SYNTAX scores indicate more extensive and complex coronary artery disease and have been associated with adverse cardiovascular outcomes in different patient populations (16–19).

Although individual components of MetS have been linked to the presence and severity of coronary artery disease, evidence regarding the relationship between the continuous MetS-Sz and the SYNTAX score is limited. To the best of our knowledge, no previous study has directly evaluated the association between these two measures in patients with chronic coronary syndrome. Therefore, the primary aim of this study was to assess whether MetS-Sz was associated with coronary artery disease complexity, as quantified by the SYNTAX score, independently of potential confounding factors in patients with chronic coronary syndrome. We hypothesized that higher MetS-Sz values would be associated with higher SYNTAX scores.

## 2 Materials and methods

### 2.1 Study design and ethical considerations

This single-center retrospective cross-sectional study was conducted using existing clinical, laboratory, and coronary angiographic data from patients diagnosed with chronic coronary syndrome. The analysis evaluated the association between MetS-Sz and angiographic coronary artery disease complexity without longitudinal assessment of subsequent cardiovascular outcomes. Electronic medical records and coronary angiography images from 2024 were reviewed. No additional examination, intervention, laboratory testing, or patient contact was performed for the purposes of the study.

The study protocol was approved by the Scientific Ethics Committee of Kanuni Training and Research Hospital, Trabzon, Türkiye (Approval No: 2024/08; Approval Date: 13 August 2024). The study was conducted in accordance with the principles of the Declaration of Helsinki. Patient data were anonymized before analysis.

### 2.2 Study population and patient selection

The electronic medical records of patients evaluated at the Family Medicine Outpatient Clinic and subsequently referred to the Cardiology Clinic during 2024 were screened. Patients diagnosed with chronic coronary syndrome who underwent coronary angiography and had the clinical and laboratory data required for calculation of the metabolic syndrome severity Z-score were eligible for inclusion.

Given the retrospective design, the sample size was determined by the number of eligible patients identified during the study period. A total of 243 patients were screened, of whom 87 were excluded: 13 did not meet the eligibility criteria relating to chronic coronary syndrome or coronary angiography; 24 had HbA1c >7%; 15 met other clinical exclusion criteria; 16 had insufficient data for MetS-Sz calculation; and 19 could not undergo SYNTAX score assessment because suitable coronary angiographic data were unavailable. The final study population comprised 156 patients. The patient selection process is summarized in Figure 1.

**Figure 1:**
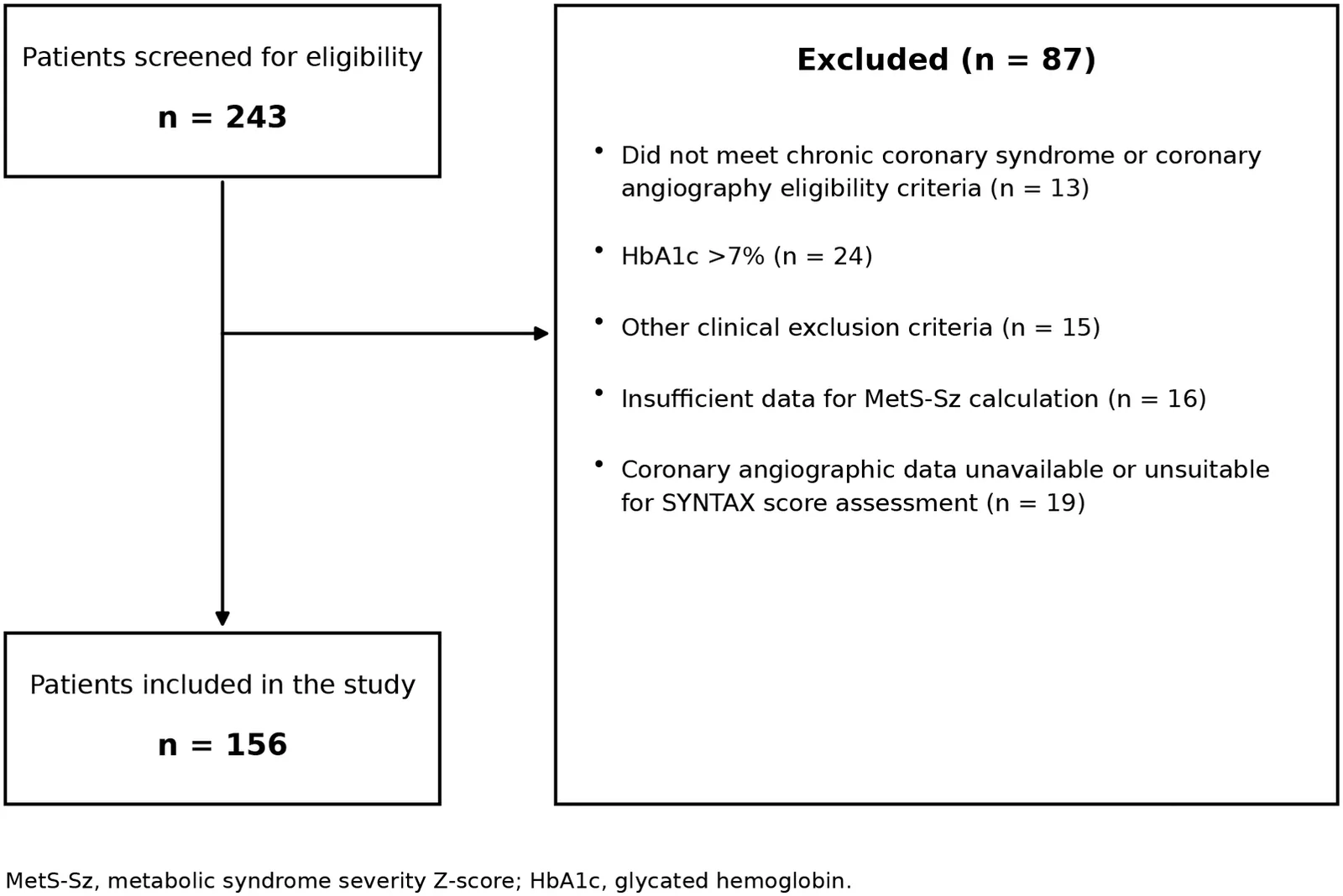
Flow diagram of patient screening and selection.

Patients with cardiogenic shock, pulmonary edema, HbA1c >7%, active infection, hematologic disease, chronic inflammatory disease, or malignancy were excluded. Age, sex, smoking status, body mass index, systolic and diastolic blood pressure, fasting glucose, HbA1c, triglyceride, total cholesterol, low-density lipoprotein cholesterol, and high-density lipoprotein cholesterol values were obtained from the electronic medical records. Existing coronary angiography images were re-evaluated for calculation of the SYNTAX score.

### 2.3 Coronary angiography

Coronary angiography had been performed in the catheterization laboratory according to standard clinical indications and contemporary guideline recommendations. The procedures were conducted predominantly via the radial approach using 6-French (F) catheters and Judkins technique.

Existing coronary angiography images were retrieved from the hospital imaging archive and re-evaluated for the purposes of the study.

### 2.4 SYNTAX score assessment

Coronary angiography images were evaluated by an experienced interventional cardiologist. Interobserver reproducibility was not assessed because independent scores from a second observer were not available. Coronary lesions with ≥50% diameter stenosis in vessels with a reference diameter of ≥1.5 mm were included in the SYNTAX score calculation. Lesions were assessed according to their anatomical location, morphological characteristics, and complexity using the original SYNTAX scoring algorithm. The score was calculated using the online SYNTAX score calculator.

Higher SYNTAX scores indicate more extensive and anatomically complex coronary artery disease. Scores of ≤22 were classified as low, scores of 23–32 as intermediate, and scores of ≥33 as high anatomical complexity.

### 2.5 The metabolic syndrome severity Z-score

The metabolic syndrome severity Z-score (MetS-Sz) was calculated using the online MetS Severity Calculator. The score provides a continuous estimate of metabolic syndrome severity based on sex- and race/ethnicity-specific equations derived from the National Health and Nutrition Examination Survey. MetS-Sz was calculated for all participants using the sex-specific equations corresponding to the non-Hispanic White category in the calculator.

The calculation included age, sex, race/ethnicity, body mass index, systolic blood pressure, fasting glucose, triglyceride, and high-density lipoprotein cholesterol values obtained from the electronic medical records. Higher MetS-Sz values indicate a greater overall burden of metabolic abnormalities.

### 2.6 Statistical analysis

Statistical analyses were performed using IBM SPSS Statistics for Windows, version 25.0 (IBM Corp., Armonk, NY, USA). The distribution of continuous variables was assessed using skewness and kurtosis values. Continuous variables were presented as mean ± standard deviation or median with interquartile range, according to their distribution, whereas categorical variables were expressed as numbers and percentages. For comparisons between two independent groups, the Student's *t*-test was used for normally distributed continuous variables and the Mann–Whitney *U*-test for non-normally distributed variables. Categorical variables were compared using two-sided Fisher's exact tests. Participants were categorized into lower (MetS-Sz < 0) and higher (MetS-Sz ≥ 0) MetS-Sz groups, corresponding to values below and at or above the reference population mean. The association between MetS-Sz and the SYNTAX score was examined using Spearman correlation analysis. Variables showing significant between-group differences and considered clinically relevant to the SYNTAX score were included in a multiple linear regression model. Multicollinearity was assessed using tolerance and variance inflation factor values. To address potential conceptual overlap between MetS-Sz and its constituent metabolic variables, an additional sensitivity analysis was conducted. This model included MetS-Sz, age, sex, and smoking status, without separately adjusting for BMI, blood pressure, glucose, triglycerides, or HDL-C. These covariates were selected on clinical grounds rather than according to between-group statistical significance. Unstandardized regression coefficients were estimated using ordinary least squares, with heteroscedasticity-consistent HC3 standard errors, 95% confidence intervals, and two-sided *p*-values. The additional analysis was performed in Python using statsmodels. Regression diagnostics included residual-vs.-fitted and normal probability plots and assessment of influential observations using Cook's distance. For the additional model, HC3 robust standard errors, an exploratory quadratic MetS-Sz term, and an influence sensitivity analysis were used to address diagnostic concerns. All tests were two-tailed, and a *p*-value < 0.05 was considered statistically significant.

## 3 Results

A total of 156 participants, including 33 women and 123 men, were included in the study. Participants were categorized into lower and higher MetS-Sz groups. Compared with the lower MetS-Sz group, the higher MetS-Sz group had significantly higher body mass index, triglyceride, total cholesterol, LDL-C, fasting glucose, diastolic blood pressure, diabetes prevalence, and SYNTAX scores, as well as significantly lower HDL-C levels. Smoking was also more frequent in the higher MetS-Sz group (all *p* < 0.05). No significant differences were observed in age, sex, systolic blood pressure, or hypertension prevalence (Table 1).

**Table 1:** Demographic, clinical, and laboratory characteristics of the participants according to MetS-Sz group.

Parameters	Lower MetS-Sz (n = 69) [−2.13—(−0.140)] [min–max]	Higher MetS-Sz (n = 87) [0.010–4.12] [min–max]	*p*-value
Anthropometric and biochemical variable			
Age (years)	63.0 ± 11.6	60.5 ± 11.6	0.178
Gender (F/M) (n)	(17/52)	(16/71)	0.430*
Smoker/non-Smoker (n)	(18/51)	(41/46)	**0**.008*
BMI (kg/m^2^)	26.0 ± 2.8	28.0 ± 3.60	**0**.**007**
TG (mg/dL)	88.0 [64.0–116]	142 [106–189]	**<0**.001**
TC (mg/dL)	191 ± 39.2	212 ± 41.6	**0**.**001**
LDL-C (mg/dL)	121 ± 39.0	157 ± 15.9	**<0**.001*
HDL-C (mg/dL)	57.7 ± 14.3	41.2 ± 9.03	**<0**.**001**
Glucose (mg/dL)	101 ± 16.6	153 ± 59.7	**<** **0**.**001**
SBP (mmHg)	112 ± 10.3	115 ± 15.2	0.086
DBP (mmHg)	66.5 ± 6.28	69.0 ± 8.96	**0**.**037**
HT (n, %)	32 (46.4)	50 (57.5)	0.198*
DM (n, %)	5 (7.2)	24 (27.6)	**0**.002*
MetS severity Z-score	−0.388 [−0.595- (−0.118)]	0.75 [0.408–1.44]	**<0.001** **
MetS severity Z-score (%) ***	34.4 ± 11.7	78.5 ± 15.8	**<0.001**
SYNTAX score	4.0 [1.05–7.0]	16.0 [9.50–21.5]	**<0.001** **

*p*-values were calculated using Student's *t*-test. Data were expressed as mean ± SD.

* *P* values for categorical variables were calculated using two-sided Fisher's exact tests.

** *p*-values were calculated using the Mann–Whitney *U*-test. Data were expressed as median (interquartile range for 25%–75%), Statistical significance was set at *p* < 0.05.

BMI, body mass index; TG, triglycerides; TC, total cholesterol; LDL-C, low-density lipoprotein cholesterol; HDL-C, high-density lipoprotein cholesterol; SBP, systolic blood pressure; DBP, diastolic blood pressure; HT, hypertension; DM, diabetes mellitus; MetS-Sz, metabolic syndrome severity Z-score.

*** MetS-Sz percentile represents the percentile value generated by the Metabolic Syndrome Severity Calculator and indicates the relative severity of metabolic syndrome compared with the reference population. Higher percentile values indicate greater cumulative metabolic burden.

Bold values indicate statistical significance (*p* < 0.05).

To further evaluate the relationship between MetS-Sz and the SYNTAX score, correlation analysis was performed. MetS-Sz showed a strong positive correlation with the SYNTAX score (*r_s_* = 0.675, *p* < 0.001), indicating that higher metabolic syndrome severity was associated with greater anatomical complexity of coronary artery disease (Figure 2).

**Figure 2:**
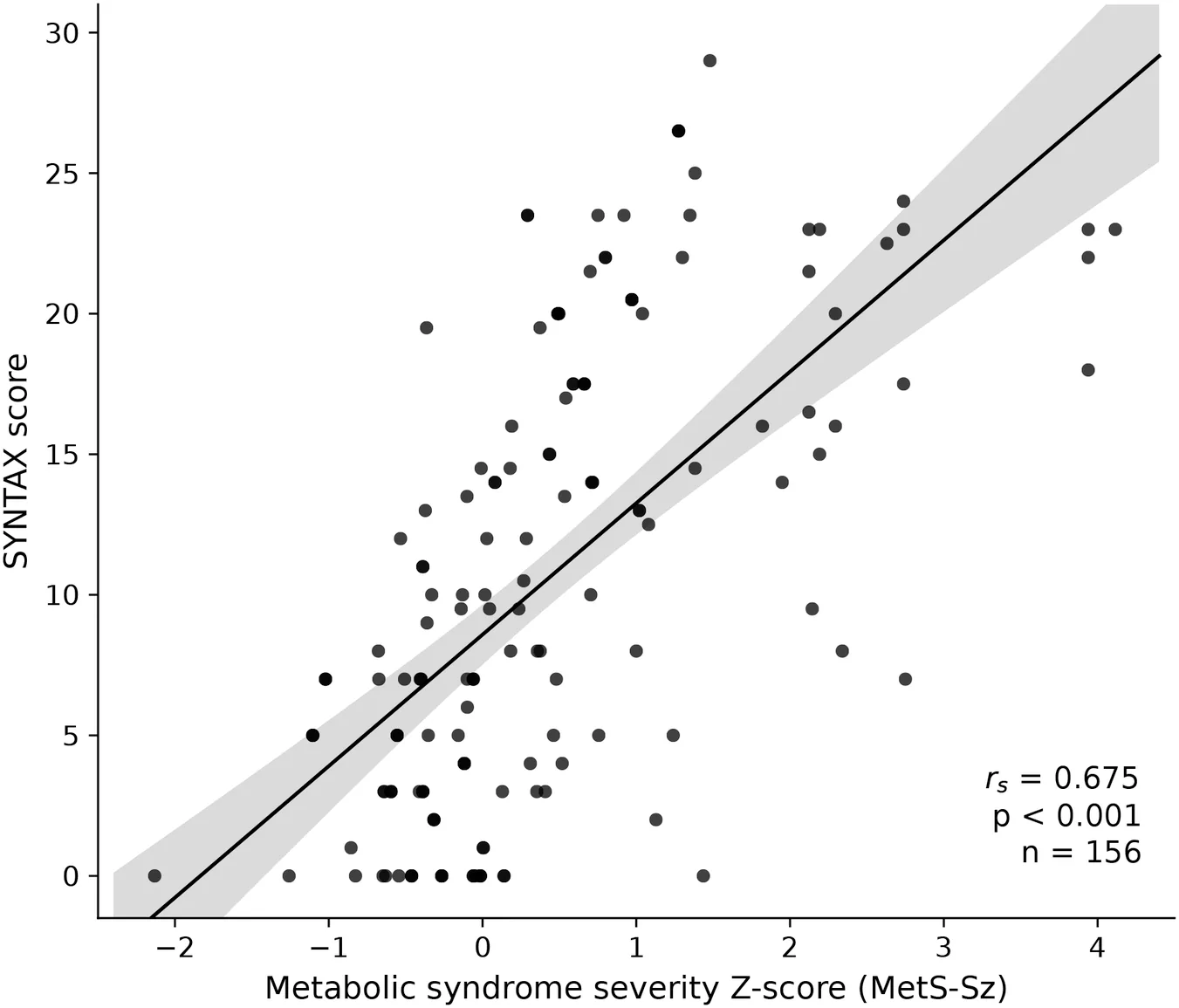
Scatter plot showing the association between the metabolic syndrome severity Z-score and the SYNTAX score. MetS-Sz was positively correlated with the SYNTAX score (*r*_s_ = 0.675, *p* < 0.001; *n* = 156).

Variables that differed significantly between the MetS-Sz groups and were considered clinically relevant to the SYNTAX score were entered into a multiple linear regression model. The overall model was statistically significant (*F* = 22.84, *p* < 0.001) and explained 51.9% of the variance in the SYNTAX score (R^2^ = 0.519). MetS-Sz, LDL-C, body mass index, triglyceride level, and smoking status were independently associated with the SYNTAX score, whereas diastolic blood pressure and diabetes mellitus were not statistically significant in the adjusted model (Table 2).

**Table 2:** Multiple linear regression analysis of factors associated with the SYNTAX score.

Coefficients^a^	Unstandardized B	SE	*t*	*p*	Tolerance	VIF
*Constant*	18.197	5.756	3.161	0.002		
MetS-Sz	6.934	.692	10.015	0.001	.370	2.702
BMI	-0.298	.149	−2.007	.047	.859	1.164
DBP	-0.015	.061	-.252	.801	.902	1.109
LDL-C	.021	.005	4.177	0.001	.753	1.328
Triglycerides	-.036	.007	−4.805	0.001	.405	2.466
DM	-.503	1.457	-.345	.730	.656	1.524
Smoking	2.053	1.005	2.043	.043	.888	1.126

a Dependent Variable: SYNTAX score; SE, Standard Error, *F* = 22.84 *p* < 0.001 R^2^ = 0.519.

B denotes the unstandardized regression coefficient, and SE denotes its standard error. For predictor variables, B represents the adjusted difference in SYNTAX score associated with a one-unit increase in a continuous variable or a change from the reference category to the comparison category for a binary variable, holding the other variables in the model constant.

b Predictors: (Constant), smoking, LDL-C, BMI, DBP, DM, triglycerides, MetS-Sz.

The overall multiple linear regression model was statistically significant (*F* = 22.84, *p* < 0.001) and explained 51.9% of the variance in the SYNTAX score (R^2^ = 0.519). MetS-Sz, LDL-C, body mass index, triglyceride level, and smoking status were independently associated with the SYNTAX score, whereas diastolic blood pressure and diabetes mellitus were not statistically significant in the adjusted model.

Among the variables included in the model, MetS-Sz showed the strongest positive association with the SYNTAX score (*B* = 6.934, *p* < 0.001). LDL-C (*B* = 0.021, *p* < 0.001) and smoking status (*B* = 2.053, *p* = 0.043) were also positively associated with the SYNTAX score. Body mass index (*B* = −0.298, *p* = 0.047) and triglyceride level (*B* = −0.036, *p* < 0.001) showed inverse associations after adjustment for the other variables. All variance inflation factor values were below 5; however, this does not exclude conceptual overlap between MetS-Sz and its constituent metabolic variables.

In the sensitivity model excluding separate adjustment for the constituent metabolic variables, MetS-Sz remained positively associated with the SYNTAX score after adjustment for age, sex, and smoking status (*B* = 4.694; HC3 95% CI, 3.812–5.576; *p* < 0.001). The model included all 156 patients and explained 43.7% of the variance in SYNTAX scores (R^2^ = 0.437; adjusted R^2^ = 0.422). The model estimates are presented in Table 3.

**Table 3:** Sensitivity analysis of the association between MetS-Sz and the SYNTAX score without separate adjustment for constituent metabolic variables.

Variable	Unstandardized B	HC3 SE	95% CI	*p*-value
Constant	6.877	3.114	0.724–13.030	0.029
MetS-Sz	4.694	0.446	3.812–5.576	<0.001
Age, years	−0.016	0.041	−0.097–0.064	0.688
Male sex vs. female sex	2.951	1.387	0.210–5.692	0.035
Smoking	1.024	1.059	−1.067–3.116	0.335

Dependent variable: SYNTAX score. *n* = 156; R^2^ = 0.437; adjusted R^2^ = 0.422. B, unstandardized regression coefficient; SE, standard error; CI, confidence interval; HC3, heteroscedasticity-consistent covariance estimator. Confidence intervals and *p*-values were calculated using HC3 standard errors. MetS-Sz, metabolic syndrome severity Z-score.

Diagnostics of the additional model indicated heteroscedasticity and departure from a linear functional form. The quadratic MetS-Sz term was significant (*p* = 0.002). Omitting six Cook-flagged observations in a sensitivity analysis retained a positive MetS-Sz coefficient (B = 5.190; HC3 95% CI, 4.302–6.079), although functional-form concerns persisted. All 156 patients were retained in the full-sample analysis.

## 4 Discussion

To the best of our knowledge, this is the first study to directly evaluate the association between the metabolic syndrome severity Z-score and the SYNTAX score in patients with chronic coronary syndrome. The principal finding was that higher MetS-Sz values were significantly associated with greater anatomical complexity of coronary artery disease. MetS-Sz showed a strong positive correlation with the SYNTAX score and remained independently associated with it after adjustment for relevant clinical and metabolic variables. The multivariable model explained 51.9% of the variance in the SYNTAX score, suggesting that cumulative metabolic burden may be closely related to coronary lesion complexity.

Previous studies have consistently shown that metabolic syndrome is associated with an increased risk of coronary artery disease, cardiovascular events, and mortality (2, 13). This relationship is thought to arise from the combined effects of abdominal obesity, insulin resistance, hypertension, atherogenic dyslipidemia, endothelial dysfunction, and chronic low-grade inflammation (13–15, 20). Unlike conventional definitions that classify metabolic syndrome categorically, MetS-Sz provides a continuous estimate of overall metabolic burden and may therefore better reflect the cumulative severity of cardiometabolic abnormalities (7). In this context, the observed association between MetS-Sz and the SYNTAX score suggests that increasing metabolic burden may also be related to the anatomical complexity of coronary atherosclerosis.

In the present study, LDL-C was positively associated with the SYNTAX score, supporting the established role of atherogenic lipoproteins in the development and progression of coronary atherosclerosis (21). The inverse adjusted associations of BMI and triglycerides require cautious interpretation because both variables contribute to MetS-Sz. Simultaneously including a composite score and its components changes the interpretation of their conditional coefficients and may produce statistical suppression, even when conventional VIF values are not elevated. These coefficients should therefore not be interpreted as evidence of protective effects. In the additional model adjusting for age, sex, and smoking without separately including the constituent metabolic variables, MetS-Sz remained positively associated with the SYNTAX score. This finding indicates that the positive association was also observed in a model without direct adjustment for the score's metabolic components, although residual confounding and model specification remain relevant considerations.

Smoking was also independently associated with a higher SYNTAX score. This finding is consistent with the well-established role of cigarette smoking in accelerating atherosclerosis through endothelial dysfunction, oxidative stress, inflammation, thrombosis, and adverse lipid changes (22, 23). The positive association observed in the adjusted model suggests that smoking may contribute to coronary lesion complexity beyond the metabolic variables included in the analysis.

Diabetes mellitus was not independently associated with the SYNTAX score in the adjusted analysis. This finding should be interpreted in the context of the study's eligibility criteria. Excluding patients with HbA1c > 7% selected a population with a restricted range of glycemic control and may have introduced selection bias, potentially attenuating or otherwise altering the observed association between diabetes and coronary artery disease complexity. However, the extent and direction of this effect cannot be determined from the present data. The absence of a statistically significant association should therefore not be interpreted as evidence that diabetes is unrelated to coronary artery disease complexity. Furthermore, diabetes was analyzed as a binary variable, which does not capture disease duration or cumulative glycemic exposure. Previous evidence indicates that glycemic variability may remain associated with cardiovascular complications even among patients who achieve conventional HbA1c targets (24).

The relationship between metabolic dysfunction and coronary artery disease may also be reflected through organ-specific manifestations. Sardjan et al. reported a significant positive correlation between the controlled attenuation parameter, an indicator of hepatic steatosis, and the SYNTAX score in patients with significant coronary artery disease (25). Although their study focused on a hepatic marker of metabolic dysfunction, our findings extend this concept by demonstrating that a composite measure of overall metabolic syndrome severity is also associated with coronary anatomical complexity. Taken together, these observations suggest that cumulative metabolic burden, whether assessed through organ-specific or integrated metabolic indicators, may provide additional information beyond individual conventional risk factors.

Anthropometric assessment beyond BMI may provide complementary information, as BMI does not capture regional body morphology or thoracic geometry. In a retrospective study of 1,091 patients undergoing exercise stress echocardiography for suspected coronary artery disease, Sonaglioni et al. assessed chest conformation using the modified Haller index (MHI), defined as the ratio of transverse to anteroposterior thoracic dimensions. A concave chest configuration, defined by MHI >2.5, was independently associated with a lower rate of cardiovascular events during follow-up (26). These observations suggest that chest geometry may provide information not captured by conventional measures of body size. However, they do not establish incremental value beyond MetS-Sz or an association with SYNTAX-defined coronary complexity. Chest geometry was not assessed in our study. Future prospective studies could examine whether combining metabolic severity scores with morphometric parameters improves cardiovascular characterization and adds prognostic information beyond conventional risk factors and angiographic disease complexity.

MetS-Sz summarizes metabolic syndrome severity using routinely available clinical and laboratory variables. The present findings support an association with angiographic coronary artery disease complexity, but do not establish diagnostic performance, prediction of future cardiovascular events, or clinical utility for risk stratification. Prospective studies with longitudinal outcomes are needed to evaluate whether MetS-Sz adds prognostic information beyond established cardiovascular risk assessment tools.

## 5 Strengths and limitations

The principal strength of this study is the simultaneous evaluation of the metabolic syndrome severity Z-score, a continuous measure of cumulative metabolic burden, and the SYNTAX score, an established angiographic measure of coronary artery disease complexity. The calculation of MetS-Sz using routinely available clinical and laboratory variables enhances the potential clinical applicability of the findings. In addition, existing coronary angiography images were re-evaluated using the standardized SYNTAX scoring algorithm.

Several limitations should be acknowledged. The single-center, retrospective cross-sectional design limits the generalizability of the findings and does not establish the temporal sequence between metabolic syndrome severity and coronary artery disease complexity. Accordingly, the observed associations should not be interpreted as evidence of causality. Furthermore, because no longitudinal cardiovascular outcomes were collected, this study cannot establish the prognostic value of MetS-Sz. The exclusion of patients with HbA1c >7% may have introduced selection bias and limits the generalizability of the findings to patients with higher HbA1c levels. This restriction should also be considered when interpreting the adjusted association between diabetes and the SYNTAX score. Additionally, MetS-Sz was calculated using equations developed for non-Hispanic White individuals in a US population. Their applicability to the Turkish study population was not specifically validated in this study, and differences between the derivation and study populations may affect score calibration and interpretation. In addition, information on potentially relevant factors, including medication use, inflammatory biomarkers, physical activity, and dietary habits, was not consistently available in the medical records. Evidence of nonlinearity limits interpretation of a single constant MetS-Sz coefficient; HC3 standard errors do not correct functional-form misspecification. SYNTAX scores were assessed by a single observer, and interobserver reproducibility could not be evaluated, leaving potential observer-related variability unquantified. Prospective multicenter studies with larger and more diverse populations are needed to confirm these findings and evaluate whether MetS-Sz provides prognostic information.

## 6 Conclusion

Higher MetS-Sz was associated with greater coronary artery disease complexity, as quantified by the SYNTAX score, in patients with chronic coronary syndrome. These findings concern an anatomical association and do not establish causality or prediction of future cardiovascular events. Prospective studies are needed to assess the clinical and prognostic relevance of this association.

## Data Availability

The raw data supporting the conclusions of this article will be made available by the authors, without undue reservation.

## References

[1] DeBoer MD. Obesity, systemic inflammation, and increased risk for cardiovascular disease and diabetes among adolescents: a need for screening tools to target interventions. Nutrition. (2013) 29(2):379–86. 10.1016/j.nut.2012.07.003 23022122 PMC3578702

[2] Alshammary AF Alharbi KK Alshehri NJ Vennu V Ali Khan I. Metabolic syndrome and coronary artery disease risk: a meta-analysis of observational studies. Int J Environ Res Public Health. (2021) 18(4):1773. 10.3390/ijerph18041773 33670349 PMC7918238

[3] Jemal A Girum T Kedir S Bedru A Mosa H Assfa K. Metabolic syndrome and its predictors among adults seeking medical care: a trending public health concern. Clin Nutr ESPEN. (2023) 54:264–70. 10.1016/j.clnesp.2023.01.034 36963872

[4] Alberti KGMM Eckel RH Grundy SM Zimmet PZ Cleeman JI Donato KA. Harmonizing the metabolic syndrome: a joint interim statement of the international diabetes federation task force on epidemiology and prevention; national heart, lung, and blood institute; American Heart Association; world heart federation; international atherosclerosis society; and international association for the study of obesity. Circulation. (2009) 120(16):1640–5. 10.1161/CIRCULATIONAHA.109.192644 19805654

[5] Grundy SM Bilheimer D Chait A Clark LT Denke M Havel RJ. Summary of the second report of the national cholesterol education program (NCEP) expert panel on detection, evaluation, and treatment of high blood cholesterol in adults (adult treatment panel II). Jama. (1993) 269(23):3015–23. 10.1001/jama.1993.03500230097036 8501844

[6] Grundy SM Cleeman JI Daniels SR Donato KA Eckel RH Franklin BA. Diagnosis and management of the metabolic syndrome: an American Heart Association/national heart, lung, and blood institute scientific statement. Circulation. (2005) 112(17):2735–52. 10.1161/CIRCULATIONAHA.105.169404 16157765

[7] Gurka MJ Lilly CL Oliver MN DeBoer MD. An examination of sex and racial/ethnic differences in the metabolic syndrome among adults: a confirmatory factor analysis and a resulting continuous severity score. Metab Clin Exp. (2014) 63(2):218–25. 10.1016/j.metabol.2013.10.006 24290837 PMC4071942

[8] DeBoer MD Gurka MJ Golden SH Musani SK Sims M Vishnu A. Independent associations between metabolic syndrome severity and future coronary heart disease by sex and race. J Am Coll Cardiol. (2017) 69(9):1204–5. 10.1016/j.jacc.2016.10.088 28254184 PMC5481721

[9] DeBoer MD Filipp SL Gurka MJ. Use of a metabolic syndrome severity Z score to track risk during treatment of prediabetes: an analysis of the diabetes prevention program. Diabetes Care. (2018) 41(11):2421–30. 10.2337/dc18-1079 30275282 PMC6196828

[10] Gurka MJ Golden SH Musani SK Sims M Vishnu A Guo Y. Independent associations between a metabolic syndrome severity score and future diabetes by sex and race: the atherosclerosis risk in communities study and jackson heart study. Diabetologia. (2017) 60(7):1261–70. 10.1007/s00125-017-4267-6 28378033 PMC5481783

[11] Motamed N Ajdarkosh H Karbalaie Niya MH Panahi M Farahani B Rezaie N. Scoring systems of metabolic syndrome and prediction of cardiovascular events: a population based cohort study. Clin Cardiol. (2022) 45(6):641–9. 10.1002/clc.23827 35419856 PMC9175260

[12] Honarvar M Mehran L Masoumi S Agahi S Khalili S Azizi F. Independent association between age-and sex-specific metabolic syndrome severity score and cardiovascular disease and mortality. Sci Rep. (2023) 13(1):14621. 10.1038/s41598-023-41546-y 37669997 PMC10480156

[13] Mottillo S Filion KB Genest J Joseph L Pilote L Poirier P. The metabolic syndrome and cardiovascular risk: a systematic review and meta-analysis. J Am Coll Cardiol. (2010) 56(14):1113–32. 10.1016/j.jacc.2010.05.034 20863953

[14] Huang PL. A comprehensive definition for metabolic syndrome. Dis Model Mech. (2009) 2(5-6):231–7. 10.1242/dmm.001180 19407331 PMC2675814

[15] Lee S-J Kim H Oh BK Choi H-I Sung K-C Kang J. Association between metabolic syndrome and left ventricular geometric change including diastolic dysfunction. Clin Cardiol. (2022) 45(7):767–77. 10.1002/clc.23838 35502633 PMC9286337

[16] Ikeno F Brooks MM Nakagawa K Kim M-K Kaneda H Mitsutake Y. SYNTAX score and long-term outcomes: the BARI-2D trial. J Am Coll Cardiol. (2017) 69(4):395–403. 10.1016/j.jacc.2016.10.067 28126156

[17] Sianos G Morel MA Kappetein AP Morice M-C Colombo A Dawkins K. The SYNTAX score: an angiographic tool grading the complexity of coronary artery disease. EuroIntervention. (2005) 1(2):219–27. 19758907

[18] Tanaka T Miki K Akahori H Imanaka T Yoshihara N Kimura T. Comparison of coronary atherosclerotic disease burden between ST-elevation myocardial infarction and non-ST-elevation myocardial infarction: non-culprit gensini score and non-culprit SYNTAX score. Clin Cardiol. (2021) 44(2):238–43. 10.1002/clc.23534 33368316 PMC7852165

[19] Esper RB Farkouh ME Ribeiro EE Hueb W Domanski M Hamza TH. SYNTAX score in patients with diabetes undergoing coronary revascularization in the FREEDOM trial. J Am Coll Cardiol. (2018) 72(23 Part A):2826–37. 10.1016/j.jacc.2018.09.046 30522646

[20] Das D Shruthi NR Banerjee A Jothimani G Duttaroy AK Pathak S. Endothelial dysfunction, platelet hyperactivity, hypertension, and the metabolic syndrome: molecular insights and combating strategies. Front Nutr. (2023) 10:1221438. 10.3389/fnut.2023.1221438 37614749 PMC10442661

[21] Borén J Chapman MJ Krauss RM Packard CJ Bentzon JF Binder CJ. Low-density lipoproteins cause atherosclerotic cardiovascular disease: pathophysiological, genetic, and therapeutic insights: a consensus statement from the European atherosclerosis society consensus panel. Eur Heart J. (2020) 41(24):2313–30. 10.1093/eurheartj/ehz962 PMC7308544 32052833

[22] Ishida M Sakai C Kobayashi Y Ishida T. Cigarette smoking and atherosclerotic cardiovascular disease. J Atheroscler Thromb. (2024) 31(3):189–200. 10.5551/jat.RV22015 38220184 PMC10918046

[23] Montero-Cabezas JM Karalis I Wolterbeek R Kraaijeveld AO Hoefer IE Pasterkamp G. Classical determinants of coronary artery disease as predictors of complexity of coronary lesions, assessed with the SYNTAX score. Neth Heart J. (2017) 25(9):490–7. 10.1007/s12471-017-1005-0 28593492 PMC5571593

[24] Ceriello A Lucisano G Prattichizzo F La Grotta R Franzén S Svensson AM. Hba1c variability predicts cardiovascular complications in type 2 diabetes regardless of being at glycemic target. Cardiovasc Diabetol. (2022) 21(1):13. 10.1186/s12933-022-01445-4 35073913 PMC8788128

[25] Sardjan J Lesmana CRA Rusdi L Kurniawan J Yunihastuti E Susilo A. Correlation between controlled attenuation parameter values with SYNTAX score in patients with significant coronary artery disease. Sci Rep. (2024) 14(1):15382. 10.1038/s41598-024-63792-4 38965252 PMC11224258

[26] Sonaglioni A Rigamonti E Nicolosi GL Lombardo M. Prognostic value of modified haller index in patients with suspected coronary artery disease referred for exercise stress echocardiography. J Cardiovasc Echogr. (2021) 31(2):85–95. 10.4103/jcecho.jcecho_141_20 34485034 PMC8388326

